# Transcranial Alternating Current Stimulation at the Individual Alpha Frequency Shows Potential Value in Improving Working Memory in Healthy Participants

**DOI:** 10.1002/brb3.71337

**Published:** 2026-05-11

**Authors:** Zhiqiang Wang, Yulan Gao, Menglin Han, Longting Hu, Kangling Wang

**Affiliations:** ^1^ Department of Rehabilitation Medicine Taikang Tongji(Wuhan) Hospital Wuhan Hubei China; ^2^ Center of Rehabilitation Medicine Zhujiang Hospital, Southern Medical University Guangzhou China; ^3^ Department of Rehabilitation The First Affiliated Hospital of Jinan University Guangzhou China; ^4^ Department of Neurological Rehabilitation Bayi Rehabilitation Center (Sichuan Rehabilitation Hospital) Chengdu China; ^5^ School of Rehabilitation Sciences Southern Medical University Guangzhou China; ^6^ GuangDong Engineering Technology Research Center of Brain Function Detection and Neuromodulation Rehabilitation Guangzhou China; ^7^ Key Laboratory of Brain Function Detection and Neuromodulation Intelligent Rehabilitation of Guangdong Higher Education Institutes Guangzhou China

**Keywords:** ERP, IAF, Neural oscillations, tACS, WM

## Abstract

Objective: To clarify the promoting effect of transcranial alternating current stimulation (tACS) at the individual alpha frequency (IAF) of the left posterior parietal cortex (PPC) on working memory (WM) using combined behavioral and electroencephalography (EEG) detection techniques. Methods: This study is a single‐blind, randomized, crossover, controlled trial with three groups: the IAF group, the IAF‐1 group, and the sham group. WM was assessed using the n‐back task, while EEG signals were simultaneously collected. Behavioral and EEG‐related indicators pre and post stimulation were compared. Results: Compared with pre‐stimulation, the average reaction time (RT) in the n‐back task significantly decreased (*p* < 0.001) and the d′ value significantly increased (*p* < 001) in all participants post‐stimulation. No significant local or whole‐brain statistical differences in event‐related desynchronization/synchronization(ERS/ERD) were found post‐stimulation. However, event‐related potential (ERP) analysis showed that the P200 amplitude (Fz: *p* = 0.023, F3: *p* = 0.017) and P300 amplitude (Fz: *p* = 0.009, Cz: *p* = 0.025) in the 3‐back task of the IAF group significantly increased after stimulation. In the IAF group, a significant negative correlation was found between ΔP300 and ΔRT (Fz: *r* = –0.465, *p* = 0.039; Cz: *r* = –0.677, *p* = 0.001). No significant correlation was found between IAF amplitude and behavioral changes (ΔRT and Δd) or ERP changes (ΔP200 and ΔP300) (*p* > 0.05). No significant correlations were found in the IAF‐1 group or the sham group (*p* > 0.05). Conclusion: IAF‐tACS specially improved the high‐load WM performance in healthy participants through increased P300 amplitude in the frontocentral region, which provides a brand‐new strategy for precise regulation in the future. Further research are needed to confirm its clinical application.

## Graphical abstract

IAF‐tACS significantly improves high‐load WM performance in healthy participants. The increased P300 amplitude in the frontocentral region during high‐load tasks suggests that parietal cortex stimulation may enhance cognitive resource allocation in the prefrontal area by modulating the frontoparietal network, which could be a key mechanism for its WM improvement.

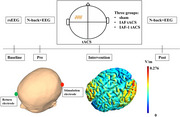

## 1 Introduction

WM is essential for higher‐order cognitive activities. Declines in WM capacity are widely observed in various neurological diseases(Alzheimer's disease (Kirova et al. 2015), Parkinson's disease (Lee et al. 2025), stroke (Liu et al. 2024), mild cognitive impairment (Jiang et al. 2025), schizophrenia (Papassotiropoulos et al. 2024), and depression (Schwefel et al. 2025.)) and normal aging adults (Bopp and Verhaeghen 2020), making it a popular target for neural regulation. tACS is a non‐invasive neural stimulation technique. By rhythmically varying the current intensity and electrode polarity, tACS induces periodic rapid depolarization and hyperpolarization of the membrane potential, thereby synchronizing neural oscillations with the exogenous stimulation to promote cognitive changes (Froehlich 2015; Bréchet et al. 2021). Numerous studies have confirmed the potential of tACS in improving WM (Reinhart and Nguyen 2019; Grover et al. 2023), with theta frequency being the most commonly used (60%), followed by gamma (22%), theta‐gamma cross‐frequency (7.5%), alpha frequency (7.5%), and beta frequency (3%) (Booth et al. 2022).

However, over the past two decades, the rapidly growing body of literature has demonstrated the effective engagement of alpha oscillations in cognitive processes, such as memory, attention, and interference suppression (Li et al. 2024; Ward 2003). This evidence has led to a paradigm shift: alpha oscillations are no longer viewed as a “passive indicator” of brain inactivity but as an active functional mechanism that regulates cognitive processing (Nolte et al. 2004; van Diepen and Mazaheri 2018; Yoshinaga et al. 2020). For instance, heightened pre‐stimulus alpha power is associated with reduced perceptual sensitivity to the stimuli (Dugué et al.), whereas attenuated alpha power is coupled with enhanced visual attention (Rajagovindan and Ding 2011). Additionally, Michels et al. (Michels et al. 2008) further demonstrated that alpha oscillatory activity is essential for maintaining stable WM representations. Given the critical role of alpha oscillations in WM and other cognitive functions, optimizing the regulation of alpha oscillations might be critical to improve cognitive performance. To achieve precise regulation of alpha oscillations, scholars have proposed the concept of individualized frequency stimulation, like using the individual's inherent alpha frequency, shortly named IAF. The theoretical basis is that the closer the tACS frequency is to the brain's inherent dominant oscillation frequency, the more extensive the synchronous activity it can induce, which is known as Arnold's tongue phenomenon (Pikovsky et al. 2002). Previous studies have shown encouraging prospects for individualized stimulation. Liu et al. 2023. found that compared with conventional 10 Hz‐tACS, IAF‐tACS can better induce alpha oscillations and effectively enhance emotional attention in healthy subjects. Cruciani et al. 2024. confirmed that tACS at individual frequency regulated more precisely the thalamo‐cortical activity when compared with tACS at a fixed‐frequency.

In view of this, our study aimed to apply IAF as the target frequency to clarify the improvement effect of IAF‐tACS on WM, and to elucidate the potential correlation between alpha oscillation and cognitive behavior. We set up three stimulation groups: IAF group, 1 Hz lower than IAF (IAF‐1) group, and the sham group. We hypothesized that compared with the sham stimulation, targeted IAF‐tACS and IAF‐1‐tACS would more effectively modulate neural oscillations individually, thereby enhancing participants' WM performance.

## 2 Methods

### 2.1 Study Design

**FIGURE 1:**
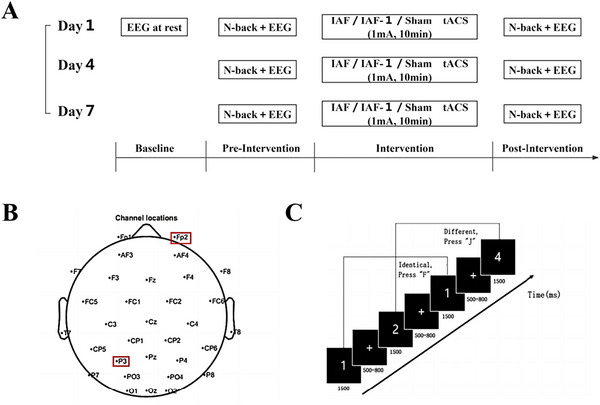
Experimental design. (A) timeline of study design; (B) example of electrode site placement.The red box shows the electrode placements;and (C) example of n‐back test with 2‐back as an example.

This study was a single‐blind, randomized, crossover, controlled trial. Each participant should accept three stimulations: IAF‐tACS, IAF‐1‐tACS, and sham‐tACS in a random order. All the stimulation trials had the same procedure, time, location, and equipment, with a 72‐h washout period set between every two stimulations. IAF was calculated personally at the very first beginning of the whole experiment through resting‐state EEG data. Then all participants were asked to perform a set of n‐back tasks (*n* = 2, 3), during which EEG data were collected. A 10‐min‐tACS stimulation came afterward, followed by another set of n‐back tasks, also with EEG data collected simultaneously. See Figure 1A for more details.

### 2.2 Participants

The inclusion criteria for participants were as follows: ① native speakers of Chinese; ② right‐handed; ③ normal vision or corrected‐to‐normal vision; and ④ aged between 18 and 30 years. The exclusion criteria were as follows: ① any acute illness requiring hospitalization within the past three months; ② history of epilepsy or any neurological or psychiatric disorders; ③ cognitive or learning disabilities; ④ history of traumatic brain injury or presence of metallic implants in the head; ⑤ history of heart disease; ⑥ pregnancy; and ⑦ history of psychoactive substance or drug use.

All participants read and signed the informed consent form before the experiment and received ¥100 RMB for completing the experiment (including 3 stimulation sessions). This study was approved by the Ethics Committee of Zhujiang Hospital, Southern Medical University, with the approval number 2023‐KY‐224‐02.

We calculated the effect size using G*Power 3.1 software. A sample size of 20 yields a power of 90% to detect an effect size of 0.43 at a significance level of 0.05 when using F‐test (ANOVA: repeated‐measures, within‐between interaction).

### 2.3 Intervention Methods

The neustim wireless neurostimulation system by Boreycon was used in our study. Two silver‐silver chloride electrodes were connected to the corresponding sites on the EEG recording cap for stimulation. The stimulating electrode was placed at the left posterior parietal location (P3 electrode), and the return electrode was placed above the right eyebrow (Fp2 electrode, see Figure 1B). All stimulation waveform were a sine wave with a relative phase of 0°, with a current intensity of 1 mA and duration time for 10 min. Conductive gel for EEG was used to ensure that the impedance remained below 10 kΩ during stimulation. Real stimulation interventions included two frequencies: IAF and IAF‐1, containing a 30 s ramp‐up and a 30 s ramp‐down period. For sham stimulation, frequency was set at IAF but current was only applied at the beginning and at the end. That is, a 30 s ramp‐up, followed by a 30 s ramp‐down at the beginning, and a 30 s fade‐in and a 30 s fade‐out at the end.

### 2.4 Task Paradigm

The WM task paradigm was programmed using EPRIME 3.0 software and presented on a fixed display screen. We employed the classic numerical n‐back paradigm with digits from 0 to 9, always consistent in font, size, color, and position. Every digit would randomly appear on the screen center after a fixation point “+” for 1500 ms, with an inter‐stimulus interval of 500–800 ms. Participants were required to determine whether the current digit on the screen was the same as the digit n positions before. If it was the same, they should press the “F” key on the keyboard as fast as possible; if it was different, they should press the “J” key as fast as possible. Our study included a set of n‐back tasks, namely 2‐back and 3‐back. In the 2‐back task, participants had to judge whether the current digit on the screen was the same as the digit two positions before (see Figure 1C for an example), while in the 3‐back task, they had to judge whether it was the same as the digit three positions before. Each task consisted of 150 trials, with 50% of the trials being target trials (same) and 50% being non‐target trials (different). A practice task was set for familiarity with the rules of the task before the formal experiment. The presentation of digits of the practice task was different from that in the formal experiment and included 30 trials which could be repeated multiple times. When the accuracy rate of the participant in the practice task reached over 80%, the formal task could be initiated.

### 2.5 Behavioral Data Analysis

The primary behavioral measures were the average RT and the d prime (d′) value. RT was calculated only from correct trials and represented the speed at which individuals responded correctly. The d′ value is an effective indicator of WM accuracy, reflecting the ability to distinguish correct responses. Compared with traditional accuracy calculations, the d′ value has the advantages of being unaffected by demographic variables or intelligence quotient (IQ) and incorporating more comprehensive data (Haatveit et al. 2010). The calculation formula for d′ is:

$$d=Z\left(\mathrm{Hit}\;\mathrm{Rate}\right)-Z\left(\mathrm{False}\;\mathrm{Alarm}\;\mathrm{Rate}\right)$$

In the formula, the hit rate represents the proportion of correct identifications among all the “same” trials, while the false alarm rate represents the proportion of incorrect identifications among all the “different” trials. The correct and incorrect trial information for each task was extracted from the EPRIME. The Z score was obtained by transforming the ratios through the Z transformation using the NORMSINV function in Microsoft Excel 2016. When participants had perfect hits or zero false alarms, the Z score would approach infinity. In such cases, a correction formula was applied:

$$\mathrm{Hit}\;\mathrm{Rate}=1-1/\left(2n\right)$$

$$\mathrm{False}\;\mathrm{Alarm}\;\mathrm{Rate}=1/\left(2n\right)$$

Here “*n*” represents the total number of the “same” or the “different” trials. “*n”* equals 75 in our experiment.

### 2.6 EEG Signal Acquisition and Data Analysis

The EEG data acquisition and analysis included both resting‐state and task‐state EEG. We used the NeuSen W series 32‐channel wireless EEG acquisition system by Boreycon. The sampling rate was 500 Hz, and the impedance of all recording electrodes was adjusted to below 20 kΩ before data acquisition. The EEG laboratory was kept quiet and with appropriate lighting. For resting‐state EEG acquisition, participants were instructed to sit comfortably with their eyes closed and to avoid any body movements for a duration of 5 min. For task‐state EEG acquisition, participants were seated in front of a 17‐inch display screen. They were instructed to adjust to a comfortable posture with their hands on the keyboard before the task began and to minimize large limb and head movements during the task.

The IAF was calculated based on the 5 min resting‐state EEG with eyes closed (Zaehle et al. 2010). Data processing was performed using the MATLAB‐based EEG analysis toolbox EEGLAB (version 2023.0). After the resting‐state EEG data were preprocessed, a fast fourier transform (FFT) was applied to identify the frequency corresponding to the highest power in the alpha band, which was used as the IAF (Rampil 1984). This study manually confirmed the IAF after calculation, and all IAF results were manually verified by two researchers with consistency over 95%.

The FFT was used to transform the time‐domain signal into the frequency domain for spectral feature extraction. The power spectral density (PSD) was calculated for each frequency band by averaging the segmented data. The specific frequency bands for analysis included delta (1–4 Hz), theta (4–8 Hz), alpha (8–12 Hz), and beta (12–30 Hz) (Koenig et al. 2001). The false discovery rate (FDR) correction was applied, and topographic maps were generated. Subsequently, the PSD was calculated for each electrode across all frequency bands.

ERPs were calculated using ERPLAB to extract the average amplitudes of the P200 and P300 components. P200 is primarily distributed in the frontal region, with target electrodes at F3, Fz, and F4 and time window from 150 ms to 275 ms post‐stimulus (Missonnier et al. 2012.). P300 is mainly distributed around the midline, with target electrodes at Fz, Cz, and Pz and time window from 300 ms to 450 ms post‐stimulus (Sowndhararajan et al. 2018). ERP data for all participants were averaged (grand average) to generate group average waveform and topographic maps for further analysis.

### 2.7 Statistical Analysis

The Shapiro‐Wilk test was used to test the normality of RT, d′, frequency amplitude at each electrode site, and PSD, while Levene's test was performed to assess the homogeneity of variances. Descriptive statistics were presented as follows: normally distributed data are reported as mean ± standard deviation (Mean±SD), and non‐normally distributed data are reported as median (interquartile range, IQR).

For within‐group analysis to characterize pre‐ and post‐stimulation changes, a paired‐samples t‐test was applied when the data met the normality assumption. The Wilcoxon signed‐rank test was used when the data did not meet the normality assumption.

A two‐way mixed‐model analysis of variance (mixed ANOVA) was performed, with group (three groups) as the between‐subjects factor and time (pre‐ and post‐intervention) as the within‐subjects factor. The model examined the main effects of group and time, and the group × time interaction effect. For the interaction effect, if the interaction was statistically significant, simple main effect analyses were conducted to compare differences between groups at each time point and changes over time within each group, so as to clarify the direction and nature of the interaction. Mauchly's test of sphericity was used to evaluate the sphericity assumption; if the sphericity assumption was violated, the Greenhouse‐Geisser correction was adopted. Post‐hoc multiple comparisons were carried out using the Bonferroni method. Partial eta‐squared (ηp^2^, defined as a measure of effect size quantifying the proportion of variance in the dependent variable explained by a specific factor or interaction) was reported for ANOVA results.

To eliminate the interference of the practice effect on the improvement of behavioral results, further analyses were conducted using ΔRT (post‐stimulation RT minus pre‐stimulation RT) and Δd′ (post‐stimulation d′ minus pre‐stimulation d′). A one‐way ANOVA was used when the data conformed to normal normality and homogeneity of variances, while the Kruskal‐Wallis test was used when the assumptions of normality or homogeneity of variances were not satisfied, with appropriate post‐hoc comparisons performed.

In other exploratory analyses, Spearman rank correlation analysis was used to evaluate the association between the changes in behavioral indicators (ΔRT, Δd′) and the changes in ERP amplitude (ΔPn) of the three groups.

All statistical analyses were performed using IBM SPSS (Version 26), and a *p*‐value < 0.05 was considered statistically significant. Statistical inferences were based on two‐tailed tests with a predefined significance level (α) of 0.05, and 95% confidence intervals (CIs) were calculated for all relevant parameters.

## 3 Results

A total of 20 healthy participants were included (10 males and 10 females), with an average age of 24.30 ± 1.22 years and mean IAF 9.88 ± 1.21 Hz. All subjects completed the trial without any dropout. Throughout the entire research process, 2 participants in the IAF group experienced a tingling sensation under the electrode as the treatment current increased, but it was mild and tolerable, and gradually subsided approximately 1 min later without affecting the experiment. Besides, 2 participants each in the IAF group and the IAF‐1 group, and 1 participant in the sham group reported transient phosphenes at the very beginning of the stimulation.

### 3.1 Behavioral Results

#### 3.1.1 2‐Back Task

RT: A significant main effect of measurement time was observed (*F* = 47.264, *p* < 0.001, ηp^2^ = 0.453), while the interaction effect between stimulation type and measurement time was not significant (*F* = 0.782, *p* = 0.462, ηp^2^ = 0.027). Bonferroni pairwise comparisons showed that post‐stimulation RT was significantly shorter than pre‐stimulation RT (547.81 ± 18.74 ms vs 602.20 ± 21.93 ms, *p* < 0.001). The Kruskal‐Wallis test showed no statistically significant difference in ΔRT among the three groups (*H* = 2.082, *p* = 0.353).

d′: A significant main effect of measurement time was observed (*F* = 15.674, *p* < 0.001, ηp^2^ = 0.216), whereas the interaction between stimulation type and measurement time was not significant (*F* = 0.102, *p* = 0.903, ηp^2^ = 0.004). Bonferroni pairwise comparisons indicated that post‐stimulation d’ value was significantly higher than pre‐stimulation d’ value (3.5 ± 0.1 vs 3.2 ± 0.1, *p* < 0.001). The Kruskal‐Wallis test demonstrated no statistical significance in Δd’ among the three groups (*H* = 0.744, *p* = 0.689).

#### 3.1.2 3‐Back Task

RT: A significant main effect of measurement time was detected (*F* = 23.051, *p* < 0.001, ηp^2^ = 0.288), and the interaction effect between stimulation type and measurement time was not significant (*F* = 0.774, *p* = 0.466, ηp^2^ = 0.026). Bonferroni pairwise comparisons showed that post‐stimulation RT was significantly shorter than pre‐stimulation RT (580.54 ± 21.23 ms vs 628.89 ± 23.24 ms, *p* < 0.001). The Kruskal‐Wallis test revealed no statistically significant difference in ΔRT among the three groups (*H* = 0.729, *p* = 0.695).

d′: A significant main effect of measurement time was observed (*F* = 15.214, *p* < 0.001, ηp^2^ = 0.211), while the interaction between stimulation type and measurement time was not significant (*F* = 0.107, *p* = 0.381, ηp^2^ = 0.013). Bonferroni pairwise comparisons indicated that post‐stimulation d’ value was significantly higher than pre‐stimulation d’ value (3.1 ± 0.1 vs 2.7 ± 0.1, *p* < 0.001). The Kruskal‐Wallis test showed no statistically significant difference in Δd’ among the three groups (*H* = 0.125, *p* = 0.927).

**FIGURE 2:**
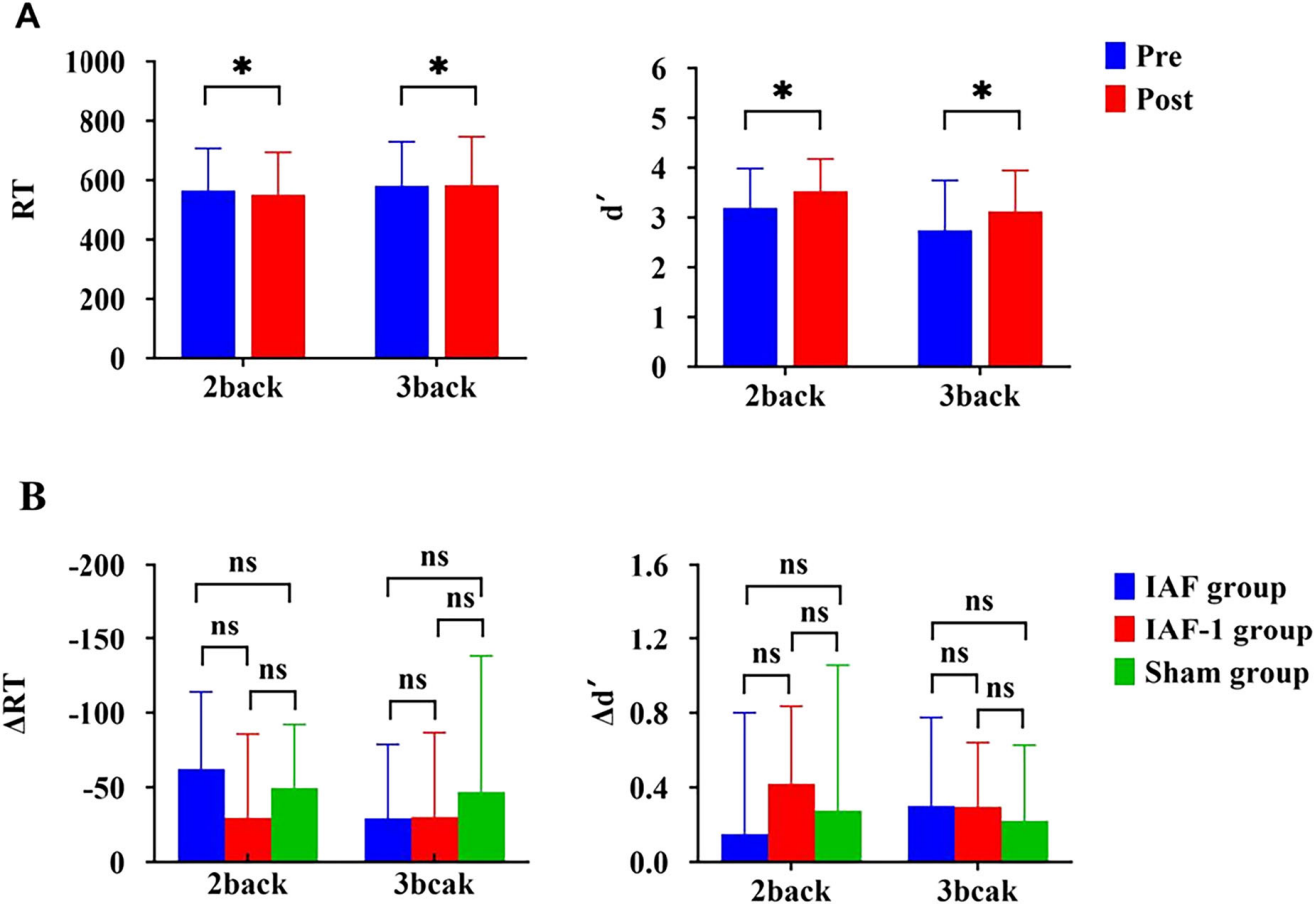
Behavioral results. A: behavioral changes pre and post stimulation in the n‐back task; B: comparison of ΔRT and Δd′ among groups; “*” represents *p* < 0.05.

All behavioral results are detailed in Table 1 and Figure 2.

**TABLE 1:** Comparison of RT and d′ pre and post stimulation in each group.

—	Group	Pre	Post	95%CI	T	P
2back‐RT(ms)	—	—	—	—	—	—
—	IAF	627.23 ± 210.66	560.01 ± 185.05	(39.98, 94.46)	5.165	<0.001*
—	IAF‐1	580.92 ± 152.36	537.77 ± 130.39	(11.42, 74.89)	2.846	0.010*
—	Sham	613.40 ± 144.42	554.38 ± 110.28	(29.82, 88.22)	4.230	<0.001*
2back‐d′	—	—	—	—	—	—
—	IAF	3.07 ± 0.88	3.36 ± 0.71	(–0.62, 0.05)	−1.758	0.095
—	IAF‐1	3.32 ± 0.60	3.67 ± 0.59	(–0.60, –0.10)	−2.892	0.009*
—	Sham	3.18 ± 0.89	3.56 ± 0.62	(–0.70, –0.050)	−2.414	0.026*
3back‐RT(ms)	—	—	—	—	—	—
—	IAF	621.25±187.80	582.52 ± 186.74	(12.76, 64.70)	3.121	0.006*
—	IAF‐1	601.09±146.38	560.82 ± 137.56	(6.36, 74.17)	2.486	0.022*
—	Sham	666.72±203.82	610.55 ± 169.59	(24.26, 118.65)	3.169	0.005*
3back‐d′	—	—	—	—	—	—
—	IAF	2.70±0.98	3.07 ± 0.91	(–0.72, –0.02)	−2.217	0.039*
—	IAF‐1	2.89±0.96	3.17 ± 0.89	(–0.53, –0.03)	−2.302	0.033*
—	Sham	2.76±0.99	3.07 ± 0.72	(–0.91, –0.06)	−2.368	0.029*

*Note*: Values are expressed as mean±standard deviation. “*” represents significant *p*‐value for within‐group comparisons pre and post stimulation.

#### 3.1.3 ERD/ERS

**FIGURE 3:**
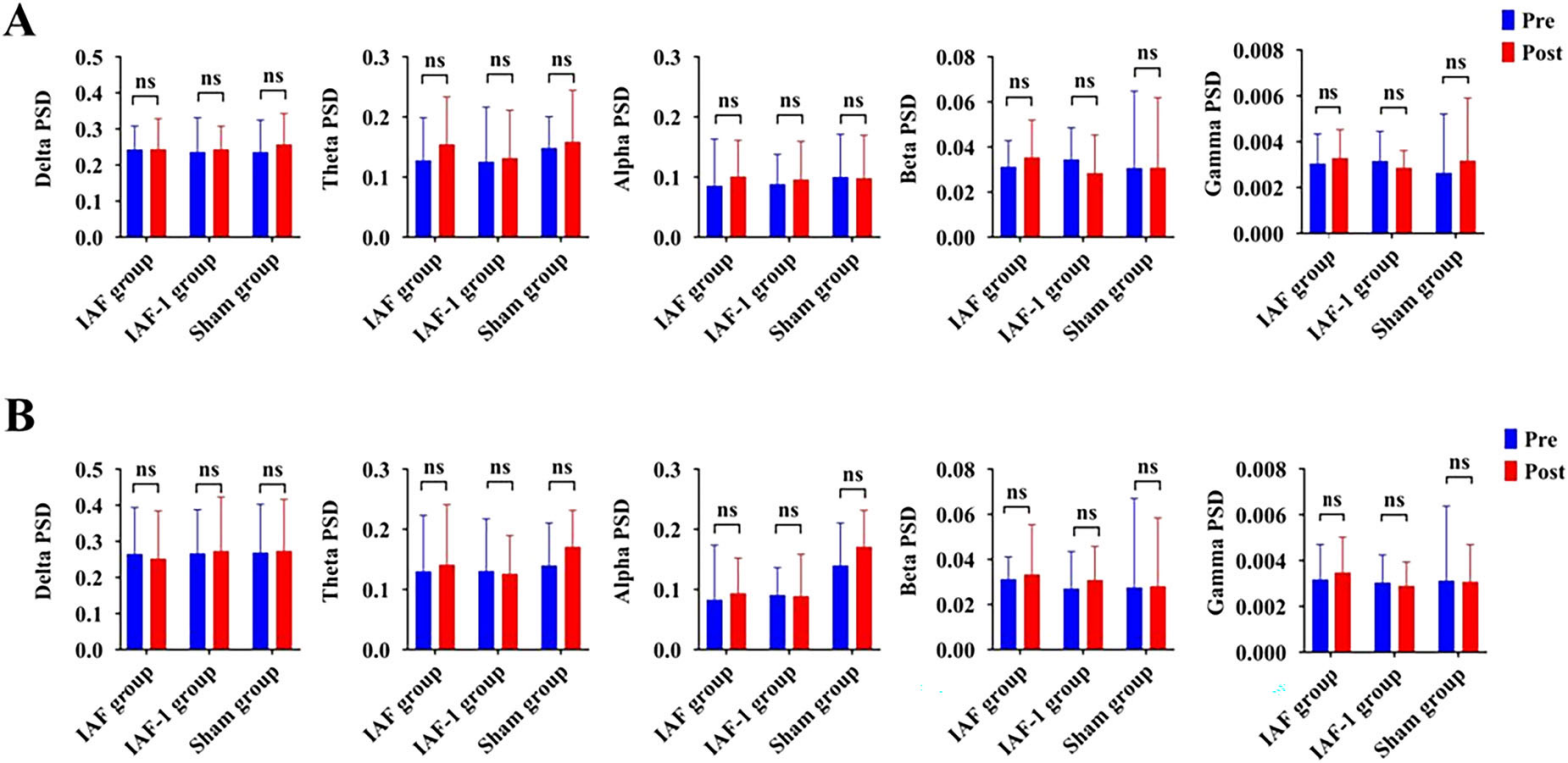
Comparison of PSD changes at the P3 electrode pre and post stimulation in the n‐back task (A, 2‐back; B, 3‐back; “ns” represents *p* > 0.05).

Given that the stimulation site was at the P3 electrode, we first extracted the local electrode data from P3, specifically the PSD of delta (δ), theta (θ), alpha (α), and beta (β) bands (µV^2^/Hz), for statistical analysis. The PSD of δ, θ, α, and β bands in both the 2‐back and 3‐back tasks did not follow a normal distribution. The Kruskal‐Wallis test was used for pre‐stimulation baseline comparisons, and the results showed no significance in either the 2‐back or 3‐back tasks. Within‐group analyses showed no significance in any task across the three groups pre and post stimulation. Between‐group analyses also showed no significant differences in the 2‐back and 3‐back tasks post‐stimulation among the three groups (*p* > 0.05, see Figure 3 for details).

**FIGURE 4:**
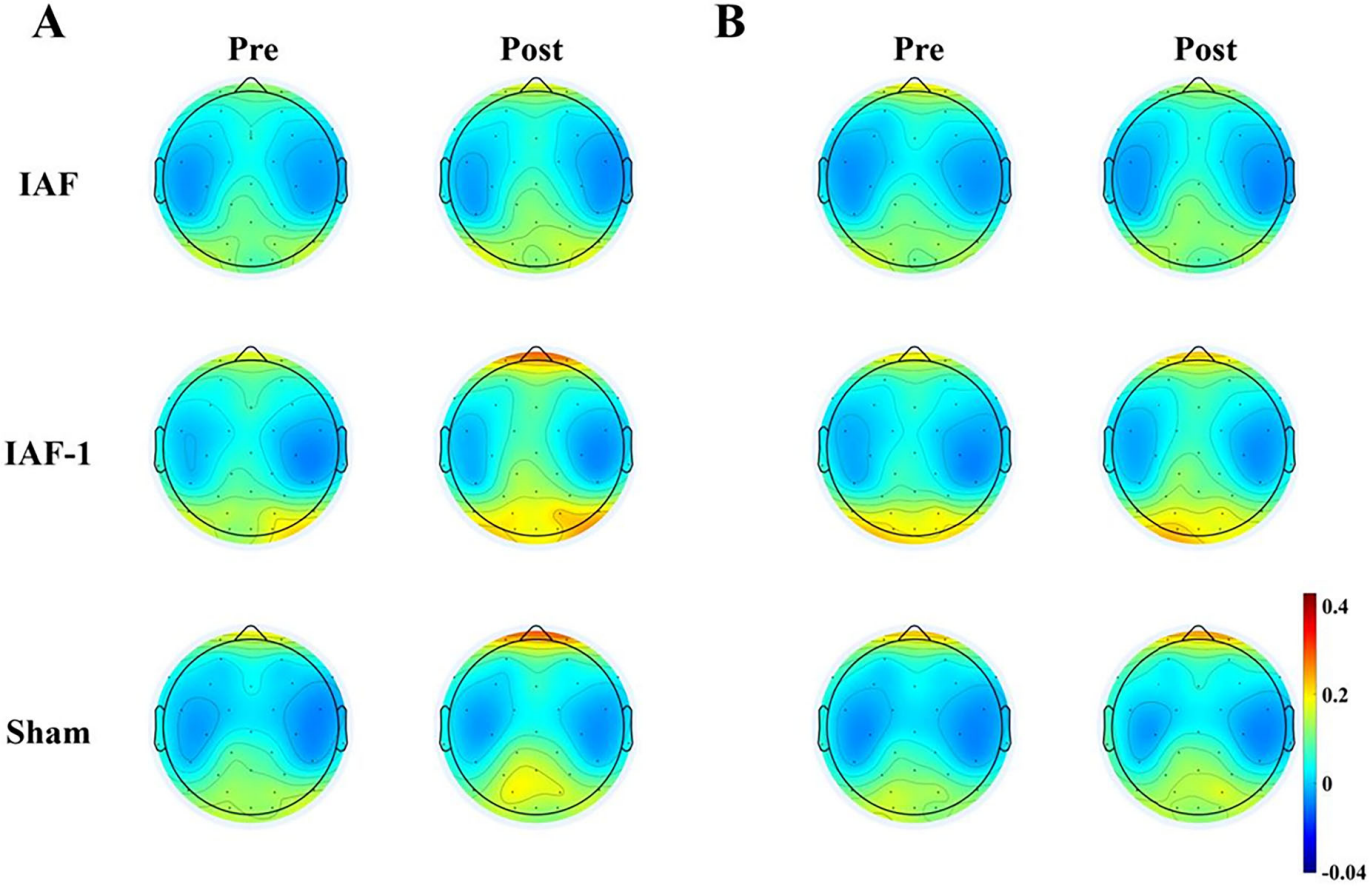
Topographic maps of PSD changes pre and post stimulation in the n‐back task (Example of the alpha band: (A) 2‐back; and (B) 3‐back).

Furthermore, we conducted statistical analyses on the PSD of δ, θ, α, and β bands across all electrodes in the whole brain. No significant differences were found within or between groups (see Figure 4 for an example of the α band).

### 3.2 ERP Results

#### 3.2.1 P200 Amplitude

Multifactorial repeated‐measures ANOVA in the 2‐back task showed a significant main effect of electrode location (*p* < 0.001), but no significant interaction was found between measurement time, stimulation type, and electrode location. Within‐group paired‐samples t‐tests revealed no significant difference between pre and post stimulation in any group.

For the 3‐back task, multifactorial repeated‐measures ANOVA showed a significant main effect of electrode location (*p* < 0.001) and a significant main effect of measurement time (*p* = 0.004). Bonferroni pairwise comparisons indicated that the amplitude post stimulation was significantly higher than that of pre stimulation (1.86 ± 0.1µV vs 1.61 ± 0.1µV, *p* = 0.004). The interaction between measurement time, stimulation type, and electrode location was not significant. Within‐group paired‐samples t‐tests showed significant increases at the F3 electrode (*p* = 0.040) after IAF stimulation, while no significant difference was observed pre and post IAF‐1 or sham stimulation.

#### 3.2.2 P300 Amplitude

Multifactorial repeated‐measures ANOVA in the 2‐back task showed no significant main effect of measurement time (*p* = 0.500), but a significant main effect of electrode location (*p* < 0.001). A significant interaction between measurement time and stimulation type was observed (*p* = 0.041). Within‐group paired‐samples t‐tests revealed a significant increase at the Pz electrode after IAF‐1 stimulation (*p* = 0.047), while no significant changes were observed in IAF or sham stimulation.

**FIGURE 5:**
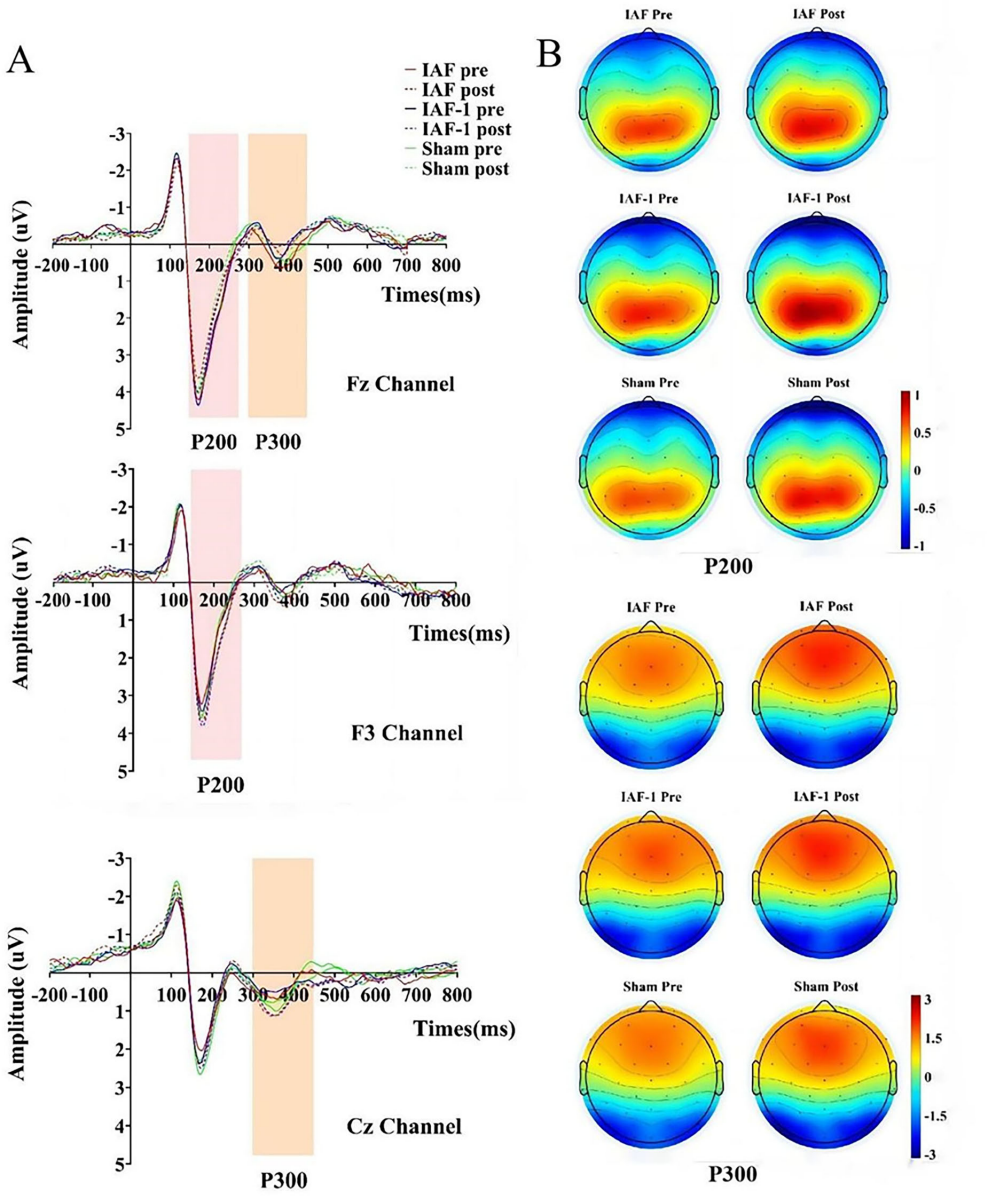
P200/P300 Waveforms (A) and Topographic Maps (B) for the 3‐back Task.

For the 3‐back task, multifactorial repeated‐measures ANOVA showed significant main effect of electrode location (*p* < 0.001) and measurement time (*p* < 0.001). Bonferroni pairwise comparisons indicated that the amplitude post stimulation was significantly higher than that of pre stimulation (0.33 ± 0.05µV vs 0.22 ± 0.05µV, *p* < 0.001). The interaction between measurement time, stimulation type, and electrode location was not significant. Within‐group paired‐samples t‐tests showed significant increases at the Fz electrode (*p* = 0.009) and Cz electrode (*p* = 0.025) after IAF‐tACS, and a significant increase at the Cz electrode after IAF‐1‐tACS(*p* = 0.044). No significant changes were observed in the sham group. Detailed ERP results are shown in Table 2 and Figure 5.

**TABLE 2:** Comparison of ERP pre and post stimulation in each group.

—		IAF(µV)				IAF‐1(µV)				SHAM(µV)			
—	—	Pre	Post	T	P	Pre	Post	T	P	Pre	Post	T	P
P200	—	—	—	—	—	—	—	—	—	—	—	—	—
Fz	2‐back	1.9 ± 1.0	2.0 ± 1.0	−1.027	0.317	1.8 ± 1.1	2.1 ± 0.9	−1.689	0.107	1.8 ± 1.0	1.8 ± 1.0	−0.351	0.729
—	3‐back	1.8 ± 1.1	2.2 ± 0.9	−2.006	0.059	2.0 ± 0.9	2.2 ± 1.0	−1.924	0.070	1.9 ± 1.0	2.1 ± 1.0	−1.753	0.096
F3	2‐back	1.4 ± 0.9	1.6 ± 1.0	−1.588	0.129	1.4 ± 1.1	1.7 ± 1.0	−1.590	0.128	1.4 ± 0.9	1.5 ± 1.0	−1.590	0.128
—	3‐back	1.4 ± 0.9	1.8 ± 0.9	−2.206	0.040*	1.5 ± 1.0	1.8 ± 1.0	−2.049	0.054	1.5 ± 0.9	1.6 ± 0.9	−1.237	0.231
F4	2‐back	1.5 ± 0.9	1.7 ± 0.7	−1.117	0.278	1.5 ± 0.9	1.7 ± 0.7	−0.757	0.458	1.3 ± 0.8	1.3 ± 0.8	−0.170	0.867
—	3‐back	1.4 ± 0.9	1.8 ± 0.8	−1.798	0.088	1.6 ± 0.7	1.7 ± 0.9	−0.575	0.572	1.4 ± 0.8	1.5 ± 0.9	−1.051	0.307
P300	—	—	—	—	—	—	—	—	—	—	—	—	—
Fz	2‐back	−0.2 ± 0.7	−0.2 ± 0.7	−0.330	0.745	−0.5 ± 0.8	−0.4 ± 0.6	−0.663	0.515	−0.3 ± 0.5	−0.4 ± 0.6	1.275	0.218
—	3‐back	−0.3 ± 0.6	−0.1 ± 0.6	−2.893	0.009*	−0.4 ± 0.6	−0.3 ± 0.6	−1.066	0.300	−0.2 ± 0.4	−0.2 ± 0.5	−0.115	0.910
Cz	2‐back	0.4 ± 0.7	0.4 ± 0.7	−0.410	0.686	0.2 ± 1.0	0.5 ± 0.7	−1.903	0.072	0.3 ± 0.8	0.2 ± 0.8	1.704	0.105
—	3‐back	0.3 ± 0.7	0.5 ± 0.7	−2.433	0.025*	0.3 ± 0.7	0.5 ± 0.6	−2.162	0.044*	0.2 ± 0.7	0.3 ± 0.7	−1.060	0.303
Pz	2‐back	0.8 ± 0.5	0.7 ± 0.6	1.820	0.085	0.6 ± 0.6	0.9 ± 0.6	−2.128	0.047*	0.8 ± 0.6	0.7 ± 0.7	0.429	0.673
—	3‐back	0.7 ± 0.6	0.7 ± 0.5	−0.622	0.541	0.7 ± 0.5	0.9 ± 0.5	−1.578	0.131	0.7 ± 0.5	0.8 ± 0.7	−0.928	0.365

*Note*: Values are expressed as mean ± standard deviation. “*” represents significant *p*‐value for within‐group comparisons pre and post stimulation.

### 3.3 Correlation Analysis Between Behavioral and EEG Changes

**FIGURE 6:**
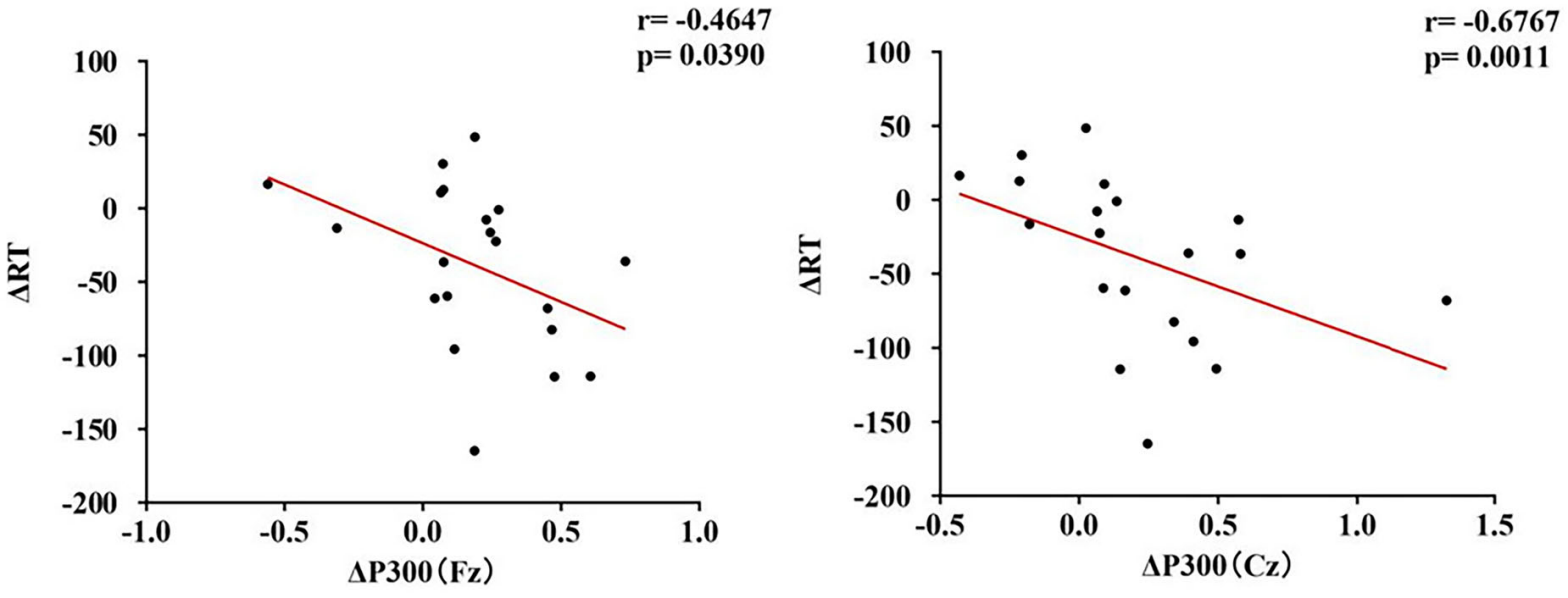
Correlation Results between ΔP300 and ΔRT in the IAF Group for the 3‐back Task.

To explore the relationship between behavioral and EEG changes, we conducted Spearman correlation analyses on the changes of RT (ΔRT = post‐stimulation RT‐ pre‐stimulation RT), d′ value (Δd = post‐stimulation d′‐pre‐stimulation d′), and ERP amplitude (ΔPn = post‐stimulation P‐wave amplitude—pre‐stimulation P‐wave amplitude). In the IAF group, during the 3‐back task, a significant correlation was found between ΔP300 and ΔRT (Fz: *r* = –0.465, *p* = 0.039; Cz: *r* = –0.677, *p* = 0.001). This indicates that the greater the increase in P300 amplitude, the greater the reduction in average RT. No significant correlations were observed in the IAF‐1 group or the sham group (all *p* > 0.05) (see Figure 6).

Additionally, we analyzed the correlation between the IAF amplitude and behavioral changes (ΔRT and Δd′) as well as ERP changes (ΔPn). No significant correlations were found in any of the three groups(all p>0.05).

## 4 Discussion

In our study, we found that all three groups had a behavioral improvement after stimulation in both RT and d', but no significance was found between groups. This does not conform to our initial hypothesis and suggests that single‐session IAF‐tACS might have no significant difference with the sham group on enhancing WM performance. We speculated that the behavioral improvements may largely lie on a learning effect of the n‐back task, for it is reported that n‐back can not only detect WM, but also improve WM ability for conductors (Schneiders et al. 2011), as well as improving other untrained cognitive functions (Pergher et al. 2018).

Although no significant difference was observed in behavioral outcomes between‐group, we specifically found an interesting EEG change that only happened in the IAF group. That is, (i) only the IAF group showed significant increases in both P200 (F3 electrode) and P300 (Fz, Cz electrodes) amplitudes after stimulation during 3‐back tasks; the IAF‐1 group only exhibited P300 elevation at the Cz electrode during 3‐back tasks; and (ii) only the IAF group revealed a negatively significant correlation between ΔP300 and ΔRT, particularly on Fz and Cz electrodes. This might suggest that the IAF‐tACS we conducted here did induce a specific EEG response.

Several points need to be stressed. First, about the classical n‐back paradigm for assessing WM (Owen et al. 2005) we used here, “*n*” represents the WM load— the higher the “*n*”, the greater the task difficulty and thus asking for higher demands on WM. Generally, 2‐back tasks are considered as medium‐load, while 3‐back tasks as high‐load. For medium load tasks, healthy participants can often successfully complete the process through effective brain recruitment without external intervention. However, when conducting high‐load tasks, the effective recruitment of the brain is limited. At this point, external intervention (e.g., tACS) can enhance participants’ task performance by activating the relevant brain regions and strengthening neural recruitment (Sazuka et al. 2024). Manza et al. also reported significant behavioral improvements after tACS in only high‐load tasks (Manza et al. 2014). This indicates that the brain is more susceptible to neuromodulation when recruitment is limited, highlighting the importance of paradigm selection that combining both the medium and high load tasks is more conducive to discover the stimulus response of tACS. Second, about P200 and P300, which are widely regarded to be closely associated with enhanced task‐related brain activation (Kim et al. 2008, Guerrero et al. 2023) and enhanced allocation of attentional resources for cognitive load, the increase of their amplitudes in our study further confirms that a single session of IAF‐tACS did lead to enhanced brain recruitment—consistent with previous research findings (Kasten et al. 2018, Clancy et al. 2018). Third, about the stimulated side of the left posterior parietal cortex (PPC), it is not only a key brain region involved in WM (Wianda and Ross 2019, Chen et al. 2023., Zeng et al. 2024), but also a primary distribution area of alpha oscillations (Chen et al. 2023., Popov et al. 2018, Minarik et al. 2018). Importantly, though numerical stimuli in the n‐back task have linguistic properties, their processing critically relies on the “spatiotemporal coding” function of the parietal lobe— a process in which alpha oscillations play a decisive role (Wianda and Ross 2019). This functional overlap between the left PPC, alpha oscillations, and n‐back task processing strongly supports the left PPC as an optimal stimulation target. Last and most, we found that not only did the P300 amplitude increase, but a significant negative correlation was also found between ΔP300 and ΔRT in the IAF group. This partially suggests a potential association between EEG responses and behavioral performance. Recently, scholars began to put forward a new viewpoint that alpha oscillations might have a causal relationship with cognition. That is, alpha power, phase, amplitude, and individual peak frequency can causally determine neural excitability, regulate signal processing in underlying neural populations, and thereby shape perception and cognition (Mathewson et al. 2012; Romei et al. 2010; Spaak et al. 2014; Trajkovic et al. 2024; Coldea et al. 2022). Trajkovic et al. (Trajkovic et al. 2024) further provided evidence through experiments revealing that the pre‐stimulation alpha amplitude of participants could predict the subsequently induced P3 amplitude, and individuals with lower alpha amplitude would exhibit higher P3 amplitude and showed stronger detection confidence during tasks. We here adopted IAF‐tACS to further explore the possibility of regulating alpha oscillations to improve cognitive behavior. To our regret, we could not found any changes in ERS/ERD of alpha oscillations or other oscillations after stimulation, either locally or globally. Additionally, no correlations were found between oscillations and P300, or between oscillations and behavioral outcomes. Therefore, this study still lacks direct evidence to confirm the causal relationship between alpha oscillations and cognitive behavior. Despite this, the correlation between ΔP300 and ΔRT remains a useful empirical evidence.

It should be noted that our study only used a single session of tACS stimulation. According to literature reports, the effects of tACS are cumulative—behavioral performance becomes more significant as the number of stimulation sessions increases (Grover et al. 2023; Agboada et al. 2025; Wischnewski et al. 2023). Therefore, based on the unique EEG response of the IAF group after stimulation, we have reason to speculate that despite the lack of significant behavioral changes in this study, the stimulation effect will become more prominent and lead to observable behavioral changes as the stimulation sessions increase. This suggests that IAF‐tACS has noteworthy potential and may well represent a novel approach for precise neuromodulation of cognitive behavior in the future. Future studies can further explore EEG responses of tACS stimulation under different stimulative sessions, different diseases, and different paradigms, to identify the optimal stimulation protocol and thereby provide more robust empirical evidence for its application in the future.

### 4.1 Limitations

Our study has some limitations. First, the sample size was relatively small. We employed a pseudo‐randomized balanced sequence for the order of stimulation for the participants, meaning that the order was randomly assigned while ensuring an equal number of participants for each stimulation sequence. This approach was adopted to avoid potential inter‐group differences in tACS stimulation effects caused by imbalances in stimulation order. Consequently, expanding the sample size would require a proportional increase to maintain overall balance. However, despite the current sample size, we observed some meaningful changes. A larger sample size would help eliminate individual variability and may reveal more interesting findings. Second, because the electrical stimulation was accompanied by a characteristic retinal phosphene, participants could easily perceive the sham stimulation, which largely compromised the blinding on the participant side. However, the blinding for IAF and IAF‐1 was successful, as participants could not distinguish the two. Third, the n‐back task was susceptible to practice effects (evident in improved performance even in the sham group), which may have masked subtle stimulation effects. This represents a design limitation, as practice effects in n‐back tasks are well‐documented and difficult to fully control with analytical approaches alone. Finally, our study only analyzed the immediate effects of a single stimulation session and did not investigate long‐term or cumulative effects. The cumulative effects of repeated stimulation are key to clinical therapeutic effects, and long‐term effects are the focus of clinical treatment. These aspects were not addressed in our study and need to be confirmed in future research.

## 5 Conclusion

This study investigated the effects of IAF‐tACS on WM tasks with different loads in healthy adults and explored the neurophysiological mechanisms underlying behavioral changes through EEG analysis. To the best of our knowledge, we for the first time reported the unique electroencephalogram effect induced by a single IAF‐tACS stimulation, revealing a potential for IAF‐tACS on cognitive regulation. Although the study did not elucidate the direct link between neural oscillations and cognitive behavior, the findings expand our current understanding of the relationship between endogenous neural oscillations and cognition and provide a new strategy for future precise modulation of cognitive behavior.

## Author Contributions

Kangling Wang and Zhiqiang Wang conceived the study. Kangling Wang and Zhiqiang Wang contributed to the study design. Zhiqiang Wang, Yulan Gao, Menglin Han, and Longting Hu collected the clinical data. Zhiqiang Wang and Yulan Gao processed the data, conducted the main analysis and wrote the original manuscript draft. Kangling Wang critically revised the manuscript and contributed the most important intellectual content. All authors revised the manuscript for important intellectual content and approved the final version.

## Funding

The authors have nothing to report.

## Ethics Statement

The study was conducted according to the guidelines of the Declaration of Helsinki, and approved by the Ethics Committee of Zhujiang Hospital, Southern Medical University,(protocol code 2023‐KY‐224‐02)

## Consent

Informed consent was obtained from all subjects involved in the study.

## Conflicts of Interest

The authors declare no conflicts of interest.

## Data Availability

The data that support the findings of this study are available from the corresponding author upon reasonable request.
